# Identification of the *OsCML4* Gene in Rice Related to Salt Stress Using QTL Analysis

**DOI:** 10.3390/plants11192467

**Published:** 2022-09-21

**Authors:** Saleem Asif, Eun-Gyeong Kim, Yoon-Hee Jang, Rahmatullah Jan, Nari Kim, Sajjad Asaf, Lubna, Muhammad Farooq, Kyung-Min Kim

**Affiliations:** 1 Department of Applied Biosciences, Graduate School, Kyungpook National University, Daegu 41566, Korea; 2 Coastal Agriculture Research Institute, Kyungpook National University, Daegu 41566, Korea; 3 Natural and Medical Science Research Center, University of Nizwa, Nizwa 616, Oman; 4 Department of Botany, Garden Campus, Abdul Wali Khan University, Mardan 23200, Pakistan

**Keywords:** QTL, salinity, Cheongcheong Nagdong double haploid, calmodulin-like protein, rice seedling

## Abstract

Soil salinity is a major abiotic stress that causes disastrous losses in crop yields. To identify favorable alleles that enhance the salinity resistance of rice (*Oryza sativa* L.) crops, a set of 120 Cheongcheong Nagdong double haploid (CNDH) lines derived from a cross between the *Indica* variety Cheongcheong and the *Japonica* variety Nagdong were used. A total of 23 QTLs for 8 different traits related to salinity resistance on chromosomes 1–3 and 5–12 were identified at the seedling stage. A QTL related to the salt injury score (SIS), qSIS-3b, had an LOD score of six within the interval RM3525–RM15904 on chromosome 3, and a phenotypic variation of 31% was further examined for the candidate genes. Among all the CNDH populations, five resistant lines (CNDH 27, CNDH 34-1, CNDH 64, CNDH 78, and CNDH 112), five susceptible lines (CNDH 52-1, CNDH 67, CNDH 69, CNDH 109, and CNDH 110), and the parent lines Cheongcheong and Nagdong were selected for relative gene expression analysis. Among all the genes, two candidate genes were highly upregulated in resistant lines, including the auxin-responsive protein IAA13 (Os03g0742900) and the calmodulin-like protein 4 (Os03g0743500-1). The calmodulin-like protein 4 (Os03g0743500-1) showed a higher expression in all the resistant lines than in the susceptible lines and a high similarity with other species in sequence alignment and phylogenetic tree, and it also showed a protein–protein interaction with other important proteins. The genes identified in our study will provide new genetic resources for improving salt resistance in rice using molecular breeding strategies in the future.

## 1. Introduction

Rice (*Oryza sativa* L.) is one of the most important cereal crops and is a basic component of diet and source of energy for more than 2.7 billion people on a daily basis [1]. It is a semiaquatic crop and a major source of food among cereal crops for more than one-half of the world’s population [2]. Besides its economic importance, it is rich in genetic diversity in the form of thousands of landraces and progenitor species. Various biotic and abiotic stresses limit its products worldwide, among which abiotic stress alone causes more than 50% of the total yield losses [3]. Soil salinity is the critical abiotic stress that limits rice production and is a major problem for rice-based farming systems in many rice-producing areas of the world [3]. At low salinity concentrations, yields are slightly changing—or not at all—but at high concentrations, yields can become zero and even sometimes result in plant death [4]. Approximately 20% of irrigated and 8% of non-irrigated agricultural land is affected by salinity [5]. It has been estimated that by 2050, salinity will affect more than 50% of arable land [6] for various reasons, including low precipitation, high surface evaporation, weathering of native rocks, irrigation with saline water, and poor cultural practices. All soils contain salts, and all irrigation waters, whether from canals or underground pumping, including those considered of very good quality, contain some dissolved salts [7]. Salinity affects plants because high salinity in soil can disturb the capacity of roots to extract water, and the presence of salts within the plant itself can be harmful, which affects many physiological and biochemical processes [3,8]. Salinity has adverse effects on plants which can cause a loss of chloroplast activity, reduced photosynthetic rate, and increased photorespiration rate, due to which a large number of reactive oxygen species (ROS) are produced [9]. The effects of salinity stress on rice may vary depending upon its different growth and development stages, starting from germination and progressing through maturation [10]. At the seedling stage, rice is susceptible to salinity, and it may cause a significant reduction in seedling vigor, seedling height, and plant biomass [1], although at the maturity stage, rice plants can tolerate salinity stress [11]. Growth reduction under salinity stress is due to the accumulation of sodium ions (Na^+^) and chloride ions (Cl^−^) in the soil, due to which the shoots and roots cannot uptake potassium ions (K^+^), calcium ions (Ca^2+^), and magnesium ions (Mg^2+^) [12,13]. The disruption in protein synthesis appears to be an important cause of damage by Na^+^ [14].

Due to genetic complexity, identifying genetic materials in response to salt tolerance is very difficult, which is why progress in rice breeding with salt tolerance remains very slow [15]. Many genes related to salinity tolerance exhibiting the quantitative or polygenic natures of the traits can be disclosed using quantitative trait locus (QTL) analysis [15,16]. QTL mapping using molecular markers can be helpful in finding salt-tolerant genes and producing salt-tolerant varieties. Different QTL analyses were conducted in different populations using molecular markers to find the salt tolerant genes. Most QTL analyses suggested that salt tolerance is a complex trait both genetically and physiologically that is controlled by polygenes, and it is reported as a polygenetic trait [17,18]. Previously, a promising QTL analysis for a salt-tolerant gene named *Saltol* was mapped on chromosome 1 at 14.7 cM using IR29/Pokkali recombinant-inbred lines (RIL) within the mapping population [19,20,21]. However, this QTL analysis alone is not enough to determine salt tolerance because this analysis did not provide tolerance in other major rice varieties. Therefore, identifying novel associated QTLs for salt tolerance would be an appropriate approach to breed new rice varieties with a high level of salt tolerance [22]. 

Ca^2+^ is an important second messenger that plays a key role in numerous biological functions, including responses to different stimuli in plants such as biotic and abiotic stresses. Different Ca^2+^ sensors or Ca^2+^ binding proteins that usually contain the “EF-hand” motif(s), a helix-loop-helix structure, can recognize the changes in concentration of Ca^2+^ [23,24]. Three major groups of Ca^2+^ binding proteins have been characterized in plants: calmodulin (CAM), calcium-dependent protein kinase (CDPK), and calcineurin B-like protein (CBL) [23,25]. CAM, which contain two pairs of Ca^2+^ binding sites, is one of the most conserved Ca^2+^ binding protein in eukaryotes [26]. Other than CAM, a large group of CAM-like genes (*CML*) has been extensively reported in plants that contain a variable number of EF-hands, having other unknown functional domains [27,28]. Different *CMLs* are significant in their responses to different abiotic stresses and have a key role in Ca^+^-mediated signaling due to their structural diversity [29]. 

In this study, Cheongcheong/Nagdong doubled haploid (CNDH) recombinant inbred lines, a cross between the Cheongcheong (*Indica*) and Nagdong (*Japonica*) lines, were used to establish an MAS system for a salt-resistant rice cultivar. The DH lines were used to map and analyze the associated genes and QTLs for different agricultural traits [30]. The main objective of this study was to detect QTLs with several traits associated with salt tolerance in rice at the seedling stage and to find associated genes by analyzing candidate genes. The results provide important information related to salt-resistant genes, which will be helpful for further cloning and for making salt-tolerant varieties.

## 2. Materials and Methods 

### 2.1. Linkage Map

A linkage map of 12 rice chromosomes was constructed from the genotypic data of the DH lines using 778 SSR markers. The linkage map covered a total genetic distance of 2121.7 cM of the rice genome, with an average interval of 10.6 cM between markers. 

### 2.2. Plant Materials and Mapping Population

In the current study, 120 CNDH lines derived from a cross between the *Indica* variety Cheongcheong and the *Japonica* variety Nagdong were used to analyze 8 different traits for salinity tolerance. The Cheongcheong/Nagdong double haploid (CNDH) line has been bred in the field at the Kyungpook National University since 2010, and it has been used for many experiments [30]. The genetic map was constructed using DH lines developed by another culture. A total of 788 SSR markers were used to build the DH lines map in which polymorphism was identified in 423 SSR markers [31]. 

### 2.3. Phenotypic Evaluation of Seedlings under Salt Stress

The 120 CNDH lines and their parental varieties at the seedling stage were provided by the plant molecular breeding lab, Kyungpook national university, and used to evaluate salt tolerance by measuring 8 different traits (shoot length (SL), root length (RL), salt injury score (SIS), chlorophyll contents (CHC), shoot fresh weight (SFW), shoot dry weight (SDW), root fresh weight (RFW), and root dry weight (RDW)). The seeds were treated with 500 µl of fungicides and soaked in water for three days at 34 °C in the incubator under dark conditions. After soaking, the pre-germinated seeds were sown in soil and kept in the dark for 3 days until successful growth was achieved. The seedlings were then transferred to 50-hole trays and, on the 21st day after planting, the seedlings were treated with 70 mM NaCl stress for one week, and then the concentration become double and 140 mM NaCl stress was given for a further 7 days. The whole experiment was conducted in a randomized complete block design with three replicates. Ten plants per line of the same growth were selected for traits related to salinity tolerance. Shoot length and root length were measured in centimeters. Shoot length was measured from the base of the culm to the tip of the tallest leaf, and root length was measured from the base of the culm to the tip of the longest root. Shoot fresh weight, shoot dry weight, root fresh weight, and root dry weight were measured in grams. 

### 2.4. Phenotypic Data Collection

After 2 weeks of salt stress, the salt injury score (SIS) was measured on the basis of a rating from 1 (highly tolerant) to 9 (highly susceptible), followed by criteria established in [20]. The chlorophyll content in five fully expanded leaves from each line was measured by a portable chlorophyll meter (SPAD 502, Konica-Minolta, Tokyo, Japan). Each leaf was measured at three points: the leaf tip, middle leaf, and leaf base. The average value was taken as the SPAD value of the leaf. Root and shoot samples were collected and washed thoroughly, and different physiological traits, including shoot length (SL), root length (RL), shoot fresh weight (SFW), shoot dry weight (SDW), root fresh weight (RFW), and root dry weight (RDW), were measured in all 120 CNDH lines and their parents. The roots and shoots were washed and dried at 70 ℃ and then measured. 

### 2.5. Statistical and QTL Analysis

The data were measured for each trait in all the CNDH lines and their parents, and different statistical analyses were carried out using the means and standard deviations. The frequency distribution, *t*-test, two-way anova, skewness, and kurtosis test were analyzed using GraphPad Prism (version 9). The Pearson’s correlation test was performed using SPSS (version 25). For gene expression analysis, two-way ANOVA was applied using Graphpad Prism (version 9). QTL analysis for the different traits was conducted using the Windows QTL Cartographer 2.5 software [32]. A composite interval mapping method was used to detect salt resistance in all traits, and an LOD of 2.0 or more was used. The R^2^ and H_1_ values were used to represent the percentage of phenotypic change and additive effects provided by the QTL mapping. All the required data including chromosome numbers, genetic distance, markers labels, genotyping data, and values of different traits were used to run the QTL analysis. The QTL naming was provided on the basis of the nomenclature proposed by McCough and Deorge [33]. 

### 2.6. Finding Candidate Genes through QTL Mapping

To find candidate genes based on QTL analysis, RiceXpro (https://ricexpro.dna.affrc.go.jp/) and RAP-DB (https://rapdb.dna.affrc.go.jp/) (accessed on 20 June 2022) were used. Candidate genes were found between the markers obtained by the QTL analysis. The functions of the candidate genes which were related to salt tolerance were found through gene ontology (GO) using the Rice Genome Annotation Project (http://rice.uga.edu/index.shtml) (accessed on 20 June 2022). NCBI (https://www.ncbi.nlm.nih.gov/) and Jalview 2.11.2.0 (https://www.jalview.org/) (accessed on 20 June 2022) were used for the multiple homologous sequences. For the phylogenetic trees, MEGA 11 (https://www.megasoftware.net/) (accessed on 20 June 2022) was used. STRING (version 11.0) (database https://string-db.org/) (accessed on 20 June 2022) was used to identify the protein–protein interactions.

### 2.7. Gene Expression Levels of Candidate Genes Related to Salinity

Expression of the related genes was checked to determine whether the related genes detected by the QTL analysis had some role in resistance to salinity. Among the CNDH population, five resistant lines (CNDH 27, CNDH 34-1, CNDH 64, CNDH 78, and CNDH 112), five susceptible lines (CNDH 52-1, CNDH 67, CNDH 69, CNDH 109, and CNDH 110), and the parent lines Cheongcheong and Nagdong were selected. These selected lines were treated with 140 mM NaCl at the seedling stage and the leaves were sampled at 0, 1, 3, 6, 12, and 24 h after the NaCl treatment for RNA extraction. RNA was extracted from the leaves using the RNeasy Plant mini kit (QIAGEN, Hilden, Germany) according to the manufacturer’s instructions. The cDNA was produced using the UltraScript 2.0 cDNA Synthesis Kit with 100 ng of RNA as a template. The qRT-PCR was performed using a qPCRBIO SYBR Green kit on an Eco Real-Time (Illumina, Singapore) machine. Approximately 20 µl of qRT-PCR reaction was used, containing 10 µl of 2 x Real-time PCR Master Mix (BioFACT, Daejeon, Korea), 7 µl RNase-free water, 1 µl of each primer, and 1 µl of template DNA. The reaction was incubated at 95 ℃ for 2 min, followed by 35 cycles at 94 ℃ for ten seconds and 60 ℃ and 72 ℃ for ten and forty seconds, respectively. The *OsActin* gene was used as a reference gene and to calculate the averages and standard deviations, and each reaction was carried out with three replicates. The primer sequences and accession numbers of all genes are provided in Table S1. 

## 3. Results

### 3.1. Phenotypic Variation

**Figure 1:**
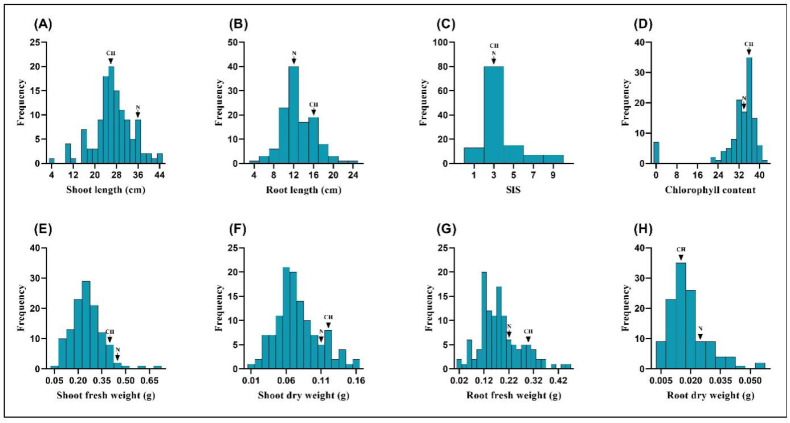
Frequency distribution of the different traits in the CNDH population (CH, Cheongcheong; N, Nagdong). All traits show a normal distribution and are controlled by polygenes. (**A**) Shoot length, (**B**) Root length, (**C**) SIS values, (**D**) Chlorophyll contents, (**E**) Shoot fresh weight, (**F**) Shoot dry weight, (**G**) Root fresh weight, and (**H**) Root dry weight.

In the present study, we evaluated the salinity tolerance of the CNDH population and their parental lines under 140 mM NaCl stress. The phenotypic distribution of the eight different traits related to salinity tolerance under 140 mM NaCl stress in 120 CNDH lines and their parents are shown in Figure 1. The mean value and standard deviation (SD) of each trait in the parents and CNDH lines, together with the *t*-test for measuring significant differences between the parents, are shown in Table S2. Eight different traits showed significant transgressive distribution, with values larger or smaller than those of the parents. In most traits, the Cheongcheong was higher than the Nagdong, showing higher resistance to salinity stress, while in SIS value, both parents were at same position and had SIS values of 3 and were considered as tolerant plants. On the basis of other traits such as SL, RL, chlorophyll content, SFW, SDW, and RFW, we could also see that both parents showed a higher resistance to salinity stress. Our results showed that both parental lines were resistant to salinity stress, and more than 80 lines among the 120 lines were resistant on the basis of their SIS values. We selected 5 resistant and 5 susceptible lines among the 120 lines and their parents’ lines for further study.

### 3.2. QTL Mapping of Traits Related to Salinity Stress

A total of 23 QTLs affecting the SL, SIS, CHC, SFW, SDW, RFW, and RDW were detected on different chromosomes, but no QTL was detected for RL, as shown in (Table 1). One QTL related to shoot length was detected on chromosome 9, having an LOD score of 2.42, between RM24288 and RM3769. The qSHL-9 showed the effect on shoot length and explained 25% of the total phenotypic variation. When the additive effect is negative its mean Nagdong alleles caused an increase in shoot length. 

A total of six QTLs related to SIS were detected on chromosomes 2, 3, 5, and 6. Out of these six QTLs, four (qSIS-2, qSIS-3a, qSIS-6a, and qSIS-6b) showed the largest effects on SIS and had phenotypic variances of 22%, 24%, 26%, and 34%, respectively. The additive effects of these four QTLs were positive, meaning that the Cheongcheong alleles contributed in these QTLs. The other two QTLs (qSIS-3b and qSIS-5) also showed a high effect on SIS and had phenotypic variances of 31% and 27%, respectively, though they both had negative additive effects, meaning that the Nagdong alleles contributed to SIS.

Five QTLs related to chlorophyll contents were identified on chromosomes 2, 5, 6, 10, and 11. Three QTLs (qCHL-2, qCHL-6, and qCHL-11) showed phenotypic variances of 23%, 21%, and 23%, respectively. The additive effects of these three QTLs were negative, meaning that the Nagdong alleles contributed to the chlorophyll contents. Similarly, the other two QTLs (qCHL-5 and qCHL-10) showed phenotypic variances of 20% and 18%, respectively, and the parent Cheongcheong alleles contributed in these two QTLs.

One QTL related to shoot fresh weight was detected on chromosome 12. This QTL, qSFW-12, showed a phenotypic variance of 22% and the parent Cheongcheong alleles contributed in this QTL.

Two QTLs related to shoot dry weight were detected on chromosome 1. One QTL, qSDW-1a, showed a phenotypic variance of 25% and had an LOD of 4.09, with the parent (Nagdong) alleles contributing in this QTL, while the other—qSDW-1b—also showed a phenotypic variance of 25% and had an LOD of 4.46, with the Nagdong alleles contributing in this QTL.

Five QTLs related to root fresh weight were detected on chromosomes 2, 7, 8, and 10. Two of these QTLs (qRFW-2 and qRFW-8a) showed phenotypic variances of 22% and 31%, respectively, and both were affected by the Nagdong alleles. The other three QTLs (qRFW-7, qRFW-8b, and qRFW-10) showed phenotypic variances of 28%, 30%, and 24%, respectively, and were affected by the Cheongcheong alleles. Among these five QTLs, two overlapped on chromosome 8 and both had a high phenotypic variance. 

**Figure 2:**
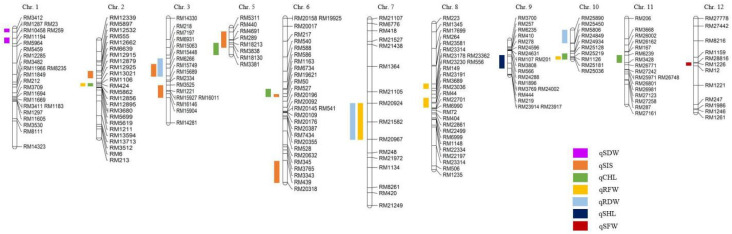
QTL mapping associated with salinity tolerance in the CNDH population. A total of 24 QTLs were detected on different chromosomes. Among all the QTLs, RM3525-RM15904 on chromosome 3 had the highest LOD scores and had salinity-resistant genes.

Three QTLs related to root dry weight were detected on chromosomes 3, 7, and 10. One QTL, qRDW-3, showed a phenotypic variance of 28%, with the parental alleles (Nagdong) contributing in this QTL. The other two QTLs (qRDW-7 and qRDW-10) showed phenotypic variances of 25% and 31%, respectively, and were affected by the Cheongcheong alleles. Finally, the genes related to salinity tolerance were screened in chromosome 3 (Figure 2).

### 3.3. Screening Candidate Genes Related to Salinity Stress on the Basis of QTL Mapping

**Figure 3:**
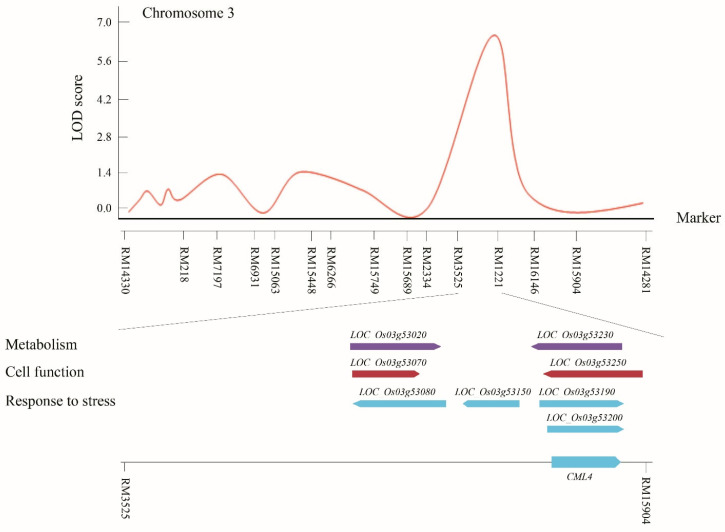
QTL analysis and physical mapping of salinity-tolerant genes. Genes associated with salinity resistance corresponding to metabolism, cell function, and stress response were identified in RM3525-RM15904 on chromosome 3. Among them, *OsCML4* plays a key role in Ca^+^-mediated signaling.

In our study, the QTL related to SIS was selected for further screening to find the candidate genes related to salinity tolerance. This QTL, qSIS-3b, had an LOD score of six within the interval RM3525–RM15904 on chromosome 3, which was screened to find candidate genes using the database (RiceXpro https://ricexpro.dna.affrc.go.jp/ (accessed on 20 June 2022)) (Figure 3). A total of 19 genes were located in this interval. This region contained the candidate genes that have the functions of tolerance and resistance, response regulation, transporter activity, kinase signaling, and flower development. Overall, a total of eight candidate genes were selected in this region based on the different sequence annotations (Table S3).

### 3.4. Relative Gene Expression of the Associated Genes in the Target Marker Region 

**Figure 4:**
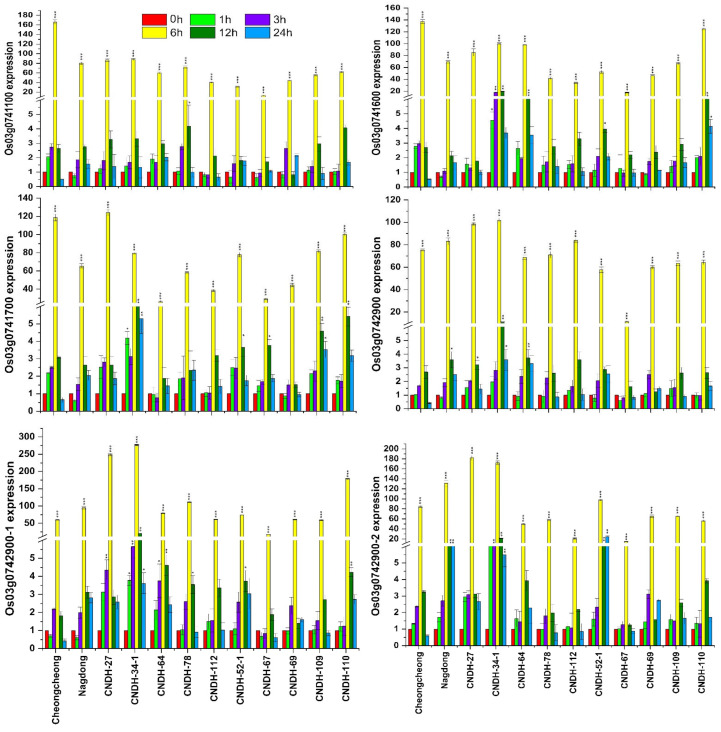
qRT-PCR results of the genes related to salinity tolerance among the Cheongcheong and Nagdong lines, as well as the five resistant and five susceptible lines. The relative expression levels of Os03g0741100, Os03g0741600, Os03g0741700, Os03g0742900, Os03g0742900-1, and Os03g0742900-2 were analyzed after 140 mM of NaCl stress at 0 h, 1 h, 3 h, 6 h, 12 h, and 24 h. Each time point was compared with 0 h in all genes. The error bars represent the standard error of each mean data (n = 3). The error bars with no asterisks indicate non-significant differences, and those with asterisks indicate a significant difference (* *p* < 0.05, ** *p* < 0.01, and *** *p* < 0.001), according to analysis using two-way ANOVA and the Bonferroni post hoc test.

**Figure 5:**
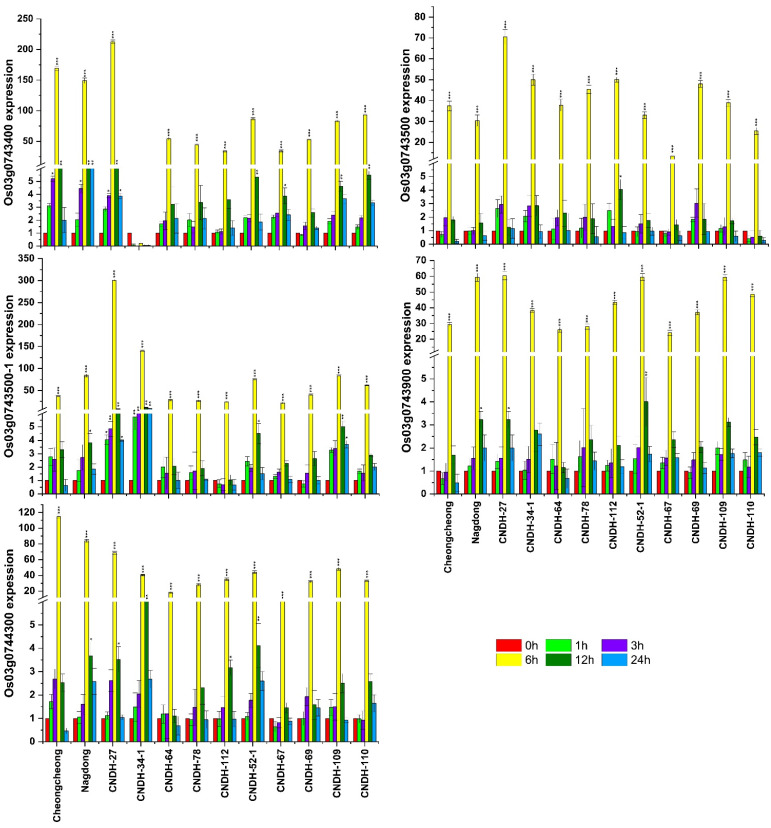
qRT-PCR results of the genes related to salinity tolerance among the Cheongcheong and Nagdong lines, as well as the five resistant and five susceptible lines. The relative expression levels of Os03g0743400, Os03g0743500, Os03g0743500-1, Os03g0743900, and Os03g0744300 were analyzed after 140 mM of NaCl stress at 0 h, 1 h, 3 h, 6 h, 12 h, and 24 h. Each time point was compared with 0 h in all genes. The error bars represent the standard error of each mean data (n = 3). The error bars with no asterisks indicate non-significant differences, and those with asterisks indicate a significant difference (* *p* < 0.05, ** *p* < 0.01, and *** *p* < 0.001), according to analysis using two-way ANOVA and the Bonferroni post hoc test.

The relative expression of salinity-associated genes was analyzed at the seedling stage. A total of eight genes (*OsbHLH148, OsPRA1*, Os03g0741700, *OsIAA13, OsH2A, OsCML4, OsATPS*, and Os03g0744300) were selected from the target region RM3525-RM15904 on chromosome 3. These eight genes were selected based on their different sequence annotations (Table S3), having the functions of tolerance and resistance, response regulation, transporter activity, kinase signaling, and flower development. Among the full CNDH population, five resistant lines (CNDH 27, CNDH 34-1, CNDH 64, CNDH 78, and CNDH 112), five susceptible lines (CNDH 52-1, CNDH 67, CNDH 69, CNDH 109, and CNDH 110), and the parent lines (Cheongcheong and Nagdong) were selected for relative gene expression analysis. All the genes in all the lines showed higher expressions after 6 h of salinity stress (Figure 4 and Figure 5). In the parent lines, all the selected genes were significantly (*p* > 0.05) expressed after 6 h, except for Os03g0743400, which consistently expressed after 3 h, 6 h, 12 h, and 24 h. The Os03g0741600, Os03g0741700, Os03g0742900, Os03g0742900-1, Os03g0742900-2, and Os03g0743500-1 genes were significantly (*p* > 0.05) expressed in the CNDH 34-1 (resistant) line after 1 h, 3 h, 6 h, 12 h, and 24 h under salinity stress. Overall, the Os03g0742900, Os03g0742900-1, Os03g0742900-2, Os03g0743500, and Os03g0743500-1 genes showed higher expression in the resistant lines, while the expression became reduced in the susceptible lines. The Os03g0743500-1 gene showed a high expression in two resistant lines (CNDH 27 and CNDH 34-1) after 1 h, 3 h, 6 h, 12 h, and 24 h of salinity stress. Among all the genes, Os03g0743500-1 (*OsCML4*), which belongs to the calmodulin-like protein (*CML*) family, was selected as the preferred gene for further study.

### 3.5. Phylogenetic Tree, Homologous Sequence, and Protein–Protein Interaction Analysis of OsCML4

**Figure 6:**
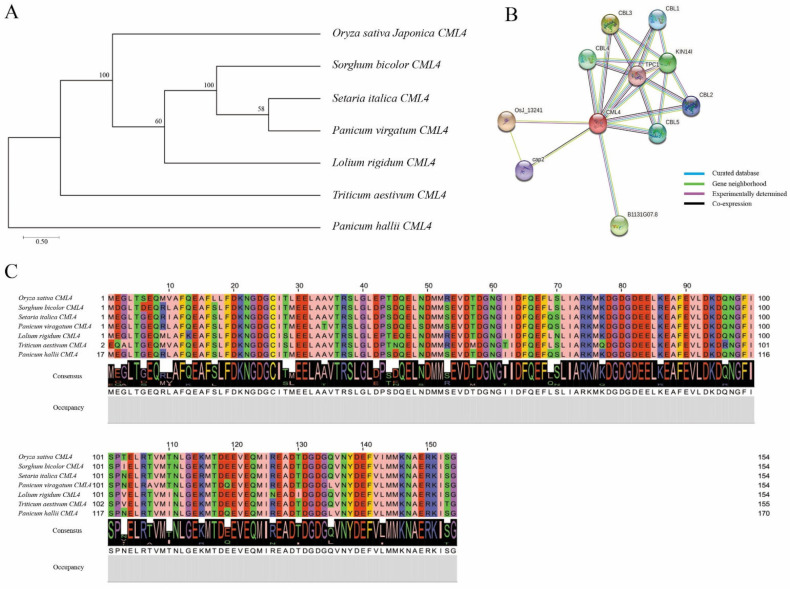
Sequence analysis of *OsCML4*. (**A**) To analyze *OsCML4* and the homology gene, a phylogenetic tree was used. (**B**) *OsCML4* interacts with CBL1, CBL2, CBL3, CBL4, CBL5, KIN14I, TPC1, OsJ_13241, cap2, and B1131G07.8. (**C**) The multiple sequence alignment of *OsCML4*; there is a high level of similarity among *Oryza sativa*, *Sorghum bicolor*, *Setaria italica*, *Panicum virgatum*, *Lolium rigidum*, *Triticum aestivum*, and *Panicum hallii*.

BLAST analysis by the NCBI database further analyzed the selected gene *OsCML4*, which is associated with salinity resistance. The phylogenetic tree shows the similarities of *OsCML4* of *Oryza sativa* to *Sorghum bicolor, Setaria italica, Panicum virgatum, Lolium rigidum, Triticum aestivum*, and *Panicum hallii* (Figure 6A). *OsCML4* was used to find the protein–protein interaction, and it was observed that *OsCML4* showed interactions with ten other proteins (CBL1, CBL2, CBL3, CBL4, CBL5, KIN14I, TPC1, OsJ_13241, cap2, and B1131G07.8) (Figure 6B). The protein sequence obtained from the NCBI database indicates that *OsCML4* of *Oryza sativa* has a very highly similar sequence to *CML4* of *Sorghum bicolor, Setaria italica, Panicum virgatum, Lolium rigidum, Triticum aestivum*, and *Panicum hallii* (Figure 6C).

### 3.6. Correlation Analysis among the Different Traits

**Figure 7:**
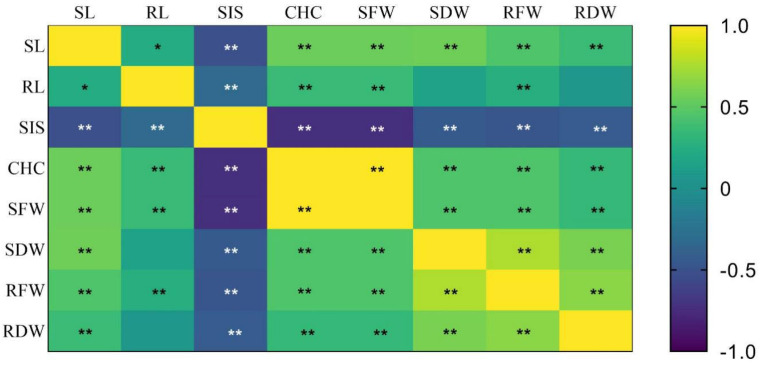
Analysis of the correlation among the different traits in the CNDH population. ** Correlation is significant at the 0.01 level (two-tailed); * correlation is significant at the 0.05 level (two-tailed).

The correlation coefficients among the different traits are as shown in Figure 7. The correlation analysis showed that all the traits were correlated with each other. According to the correlation analysis, there is a positive correlation among SL, RL, CHC, SFW, SDW, RFW, and RDW, while the SIS had a negative correlation with all the other traits. The SIS showed a highly significant negative correlation with SL (r = −0.52), RL (r = −0.32), CHC (r = −0.73), SFW (r = −0.73), SDW (r = −0.45), RFW (r = −0.47), and RDW (r = −0.42). CHC and SFW were very strongly related, having a coefficient of r = 1.00.

## 4. Discussion

Salinity has been a major problem for rice production for soil is under the threat of salinity. Rice grain yields have been reduced by up to 100% depending on two factors: the level of salinity stress and the duration of time the rice plants are exposed to saline conditions [34]. Previously, it was reported that there were 14 QTLs related to salt tolerance on chromosomes 2, 3, 5, 6, 8, and 9, using a set of F2 populations derived from a cross between a salt-tolerant Tarommahali (TAM) and a salt sensitive Khazar (KHZ) [35]. In our study, we used 120 CNDH lines derived from a double haploid cross between the *Indica* variety Cheongcheong and the *Japonica* variety Nagdong, and we identified a total of 23 QTLs for salt tolerance on different chromosomes. A comparison of the QTLs related to salt tolerance in our study with those reported by [35] showed that QTL *QSIS-3*, mapped near RM3525 on chromosome 3, shared a similar chromosomal region with three QTLs (*qSTR-3b, qNAK-3*, and *qNA-3*) from [35], and *qSIS-2*, *qRFW-2*, and *qCHL-2*, detected on chromosome 2 near RM1106, were located in a similar region with a QTL (*qNA-2a*) reported by [35]. 

Previously, many QTLs have been detected in *O. sativa* populations. The results showed that *qST5-1* near RM3328 on chromosome 5 was in a similar location to *qDLRa5-2* and *QSst5a* [36,37], and *qST5-2* was located in a similar location to *qDSRs5-1, QDss5, QSf5*, and *QGW5* [37,38], further confirming the reliability of the salt stress-related QTLs detected in the present study. Moreover, the results indicated that the stable QTLs might be useful for rice breeding using marker-assisted selection, accelerating the development of salt-tolerant rice varieties.

We compared our QTL results with other reported results. The results showed that qSIS-5 near RM4691 on chromosome 5 was in similar location to qST5-2; qCHL-10 and qRFW-10, near RM25219 on chromosome 10, were in similar location to qST10 on chromosome 10; and qSFW-12 near RM1226 on chromosome 12 was in a similar location to qST12 on chromosome 12 [39]. 

From the QTL mapping and relative gene expression analysis of the eight genes located in RM3525-RM15904 on chromosome 3, two candidate genes were colocalized with QTL for salinity resistance, including the auxin-responsive protein IAA13 (Os03g0742900) and the calmodulin-like protein 4 (Os03g0743500-1). Overall, both the auxin-responsive protein IAA13-like (Os03g0742900) and the calmodulin-like protein 4 (Os03g0743500-1) showed higher expression in all the resistant lines than in the susceptible lines. Auxin/IAA are short-lived proteins that regulate cell division and elongation to drive plant growth and developmental processes [40,41]. A number of auxin/IAA gene families have been identified in many plants, including arabidopsis [42], rice [43], maize [44], and chickpea and soybean [40]. In rice, a total 31 of auxin/IAA genes have been identified which are induced by salt, drought, and other osmotic stresses [41]. *OsIAA13* in rice is involved in lateral root initiation [45]. In arabidopsis, a total of 29 auxin/IAA genes have been identified [42]. The gene Os03g0742900 may function in response to salt stress through its the expression.

Our main focus is on *OsCML4*, the calmodulin-like protein located in the QTL region. In arabidopsis, many CMLs are induced under different biotic and abiotic stress conditions. *CML24*, a calmodulin-like gene, shows responses to various salt stresses [46,47]. In rice, several *OsCML* genes are highly expressed in plants’ organs and tissues, and their expression varies in different developmental stages. *OsCML1, 4, 5, 8*, and *11* were highly expressed in rice under salinity stress (150 mM NaCl), suggesting that these genes may function in the mechanisms of a Ca^2+^-mediated response to salinity [27]. In our study, the *OsCML4* gene is significantly (*p* > 0.05) expressed under a salinity stress of 140 mM, showing higher expression in the CNDH resistant lines and lower expression in the CNDH susceptible lines. Additional research on *OsCML4* will be helpful for identifying the mechanisms of salinity stress and for developing cultivars that are salt resistant. Our studies also revealed that *OsCML4* has a very similar sequence to *CML4* in *Sorghum bicolor, Setaria italica, Panicum virgatum, Lolium rigidum, Triticum aestivum*, and *Panicum hallii*. The CMLs are widely involved in different developmental and stress tolerance mechanisms in different plants species [48]. The protein–protein interaction also revealed that *OsCML* interacted with ten other proteins (CBL1, CBL2, CBL3, CBL4, CBL5, KIN14I, TPC1, OsJ_13241, cap2, and B1131G07.8) (Figure 6b). The CBLs are calcineurin B-like proteins which are important Ca2+ sensors in calcium signaling pathways and involved in salinity resistance and other abiotic stress responses [49]. 

## 5. Conclusions

This study concluded that plants show tolerance to stress conditions by regulating their gene expression. In the present study, QTL analysis was performed in response to salinity stress at the seedling stage. In the QTL mapping results, a total of 23 QTLs for eight traits were mapped. One QTL related to SIS was screened at RM3525-RM15904, and eight candidate genes related to salinity tolerance were identified. Among the eight genes, only *OsCML4* was selected as a candidate gene because the database-based study showed that this gene has the functions of response regulation, transporter activity, and tolerance and resistance to salinity stress. After the application of salinity stress, the relative gene expression of *OsCML4* was higher in the resistant lines (CNDH-27 and CNDH-34-1) than in the susceptible lines. This gene can be used for further studies for developing rice cultivars that show resistance to salinity stress.

## Figures and Tables

**Table 1:** QTLs associated with salinity tolerance in the CNDH population.

Characteristics	QTLs	Chr	Interval Markers ^z^	LOD	Additive effect ^y^	R^2 x^	Increasing effects ^w^
Shoot length	qSHL-9	9	RM24288-RM3769	2.42	−2.61	0.25	Nagdong
SIS	qSIS-2	2	RM6639-RM12879	2.76	0.66	0.22	Cheongcheong
	qSIS-3a	3	RM15448-RM15749	2.77	0.65	0.24	Cheongcheong
	qSIS-3b	3	RM3525-RM15904	6	−0.98	0.31	Nagdong
	qSIS-5	5	RM440-RM18130	2.5	−0.53	0.27	Nagdong
	qSIS-6a	6	RM19621-RM50	2.13	0.48	0.26	Cheongcheong
	qSIS-6b	6	RM439-RM20318	2.44	0.73	0.34	Cheongcheong
Chlorophyll content	qCHL-2	2	RM1106-RM5862	2.36	−2.89	0.23	Nagdong
	qCHL-5	5	RM18213-RM3381	2.45	2.64	0.2	Cheongcheong
	qCHL-6	6	RM1163-RM50	2.17	−2.75	0.21	Nagdong
	qCHL-10	10	RM25128-RM25036	2.3	2.64	0.18	Cheongcheong
	qCHL-11	11	RM6239-RM3428	2.29	−2.63	0.23	Nagdong
Shoot fresh weight	qSFW-12	12	RM28816-RM12	2.3	29.98	0.22	Cheongcheong
Shoot dry weight	qSDW-1a	1	RM3412-RM1287	4.09	−15.14	0.25	Nagdong
	qSDW-1b	1	RM10458-RM11194	4.46	15.49	0.25	Cheongcheong
Root fresh weight	qRFW-2	2	RM1106-RM5862	2.36	−2.84	0.22	Nagdong
	qRFW-7	7	RM20924-RM20967	4.36	31.51	0.28	Cheongcheong
	qRFW-8a	8	RM23314-RM23178	2.14	−26.57	0.31	Nagdong
	qRFW-8b	8	RM23191-RM44	2.51	27.32	0.3	Cheongcheong
	qRFW-10	10	RM25219-RM25036	2.22	2.75	0.24	Cheongcheong
Root dry weight	qRDW-3	3	RM15063-RM15749	3.52	−3.83	0.28	Nagdong
	qRDW-7	7	RM20924-RM20967	3.44	3.36	0.25	Cheongcheong
	qRDW-10	10	RM25890-RM5806	3.07	4.42	0.31	Cheongcheong

Note: ^z^—the markers which are in the significance threshold; ^y^—the positive values indicate the contribution from the mother plant; ^x^—the phenotypic variation; and ^w^—the source of the allele generating an increase in the assessed traits.

## Data Availability

The data presented in this study are available on request from the corresponding author.

## Supplementary Materials

The following supporting information can be downloaded at: https://www.mdpi.com/article/10.3390/plants11192467/s1, Table S1: Primers and accession numbers used for qRT-PCR. Table S2: Phenotypic values of different traits. Table S3: Genes related to salinity tolerance were screened from the interval RM3525-RM15904 on chromosome 3.
