# Supplementary material for: Identification of the OsCML4 Gene in Rice Related to Salt Stress Using QTL Analysis
Source: Plants (Basel). 2022 Sep 21;11(19):2467. doi: 10.3390/plants11192467 (PMC9572784; doi:10.3390/plants11192467)
Supplement: Supplementary file 1 [file plants-11-02467-s001.zip › plants-1898258-supplementary.pdf]

Supplementary data

Supplementary tables

Table S1: Primers and accession numbers used for qRT-PCR.

Locus	Accession no	forward primer	reverse primer
Actin	XM 015785964.2	TGAATCTGGTCCAGGCATCG	TGGGACGCATGCAAACAATC
Os03g0741100	AK071734	GGAAACCATCGCCAACCATC	CGACTGCACGATCGAGTTCT
Os03g0741600	AC096855	TCCGGCAACCATAGTGTTCC	CCCGATGAGCAAACCTTCCT
Os03g0741700	CI363564	TGATCTTGGCTGTGATCCTG	ACCCTCGGAGTGTCAACAAC
Os03g0742900	AK062852	GATGTCCCGTGGCAGATGT	AAGACCAATGGCTTCGGAAC
	AK100790	ACCCGCAACTGGGAACAATA	CTCGACAAACATCTGCCACG
	AJ251791	GGGCTTCGAGGAGACCATTG	GGCTTCGGAACCCTTCATGA
Os03g0743400	AK120299	AGGACTCAGGCAAATGGACG	TAACTGCAGAGACCAGGCAG
Os03g0743500	AK069620	GTTCATGTGTGCAGCTGAGG	ATCGGTATCCGCCTCCCTTA
	AK067697	TTCCTGAGCCTCATTGCCAG	TCGGTATCCGCCTCCCTTAT
Os03g0743900	AK099593	TTGCGGCCTAGAAAGCTGAA	GCGCTTCCAATCAAACCCAA
Os03g0744300	AK102540	TTGATGAGACAGCCAGCCAG	GCCAGATCTGTTGACCTGCT

Table S2: Phenotypic values of different traits.

Traits	Parents			
	Cheongcheong Mean ± SD	Nagdong Mean ± SD	CNDH Mean ± SD	t-value ($P_1 – P_2$)
Shoot length (cm)	25.33 ± 0.57	37.33 ± 1.52	26.35 ± 7.13	0.0002^{***}
Root length (cm)	14.86 ± 1.20	11.23 ± 0.68	12.71 ± 3.33	0.0105^{*}
SIS	5 ± 0.96	3 ± 1.032	4.022 ± 1.75	0.5185^{ns}
Chlorophyll content	35.9 ± 1.51	34 ± 1.50	32.03 ± 8.82	0.1985^{ns}
Shoot fresh weight (g)	0.34 ± 0.013	0.24 ± 0.015	0.15 ± 0.06	0.0075^{**}
Shoot dry weight (g)	0.072 ± 0.007	0.06 ± 0.006	0.045 ± 0.018	0.0309^{*}
Root fresh weight (g)	0.168 ± 0.003	0.13 ± 0.004	0.109 ± 0.05	0.0001^{***}
Root dry weight (g)	0.006 ± 0.003	0.012 ± 0.003	0.011 ± 0.005	0.0309^{*}

Table S3: Genes related to salinity tolerance were screened from the interval RM3525-RM15904 on chromosome 3.

**Function**	**Locus**	**Description**	**No of genes**
Biosynthetic process.	LOC_Os03g53020	Basic helix-loop-helix dimerisation region bHLH domain containing protein.	1
Molecular function.	LOC_Os03g53070	Prenylated rab acceptor PRA1 family protein.	1
Response to stress.	LOC_Os03g53080	Zinc finger, RING-type domain-containing protein.	1
Biosynthetic process.	LOC_Os03g53150	Aux /IAA protein.	4
Response to stress.	LOC_Os03g53190	Similar to H2A protein.	1
Response to stress	LOC_Os03g53200	Similar to Calmodulin 1 (Fragment).	2
Metabolic process.	LOC_Os03g53230	Similar to ATP sulfurylase.	1
Cellular component.	LOC_Os03g53250	Protein kinase-like domain-containing protein.	1
